# Delivery decision in pregnant women rescued by ECMO for severe ARDS: a retrospective multicenter cohort study

**DOI:** 10.1186/s13054-022-04189-5

**Published:** 2022-10-17

**Authors:** Sarah Aissi James, Christophe Guervilly, Mathieu Lesouhaitier, Alexandre Coppens, Clément Haddadi, Guillaume Lebreton, Jacky Nizard, Nicolas Brechot, Benjamin Assouline, Ouriel Saura, David Levy, Lucie Lefèvre, Pétra Barhoum, Juliette Chommeloux, Guillaume Hékimian, Charles-Edouard Luyt, Antoine Kimmoun, Alain Combes, Matthieu Schmidt

**Affiliations:** 1 Service de Médecine Intensive-Réanimation, Institut de Cardiologie, APHP Sorbonne Université Hôpital Pitié-Salpêtrière, 75013 Paris, France; 2 Service de Médecine Intensive-Réanimation, APHM, CHU Hôpital Nord, Marseille, France; 3 Service de Maladies Infectieuses Et Réanimation Médicale, CHU de Rennes, Rennes, France; 4 CHRU de Nancy, Institut Lorrain du Cœur Et Des Vaisseaux, Service de Médecine Intensive-Réanimation, U1116, FCRIN-INICRCT, Université de Lorraine, Nancy, France; 5 Sorbonne Université, INSERM, UMRS_1166-ICAN, Institute of Cardiometabolism and Nutrition, F-75013 Paris, France; 6 Service de Chirurgie Cardiaque, Institut de Cardiologie, APHP Sorbonne Université Hôpital Pitié-Salpêtrière, 75013 Paris, France; 7 Department of Gynaecology and Obstetrics, Groupe Hospitalier Pitié-Salpêtrière, CNRS UMR 7222, INSERM U1150, Sorbonne Universités, Paris, France; 8 Sorbonne Université, GRC 30, RESPIRE, Assistance Publique-Hôpitaux de Paris (APHP) Hôpital Pitié-Salpêtrière, Paris, France; 9 Service de Medecine Intensive Reanimation, iCAN, Institute of Cardiometabolism and Nutrition, Hôpital de La Pitié-Salpêtrière, 47, Bd de L’Hôpital, 75651 Paris Cedex 13, France

**Keywords:** Extracorporeal membrane oxygenation, Pregnant women, Acute respiratory distress syndrome, Delivery, Outcomes

## Abstract

**Background:**

Although rarely addressed in the literature, a key question in the care of critically pregnant women with severe acute respiratory distress syndrome (ARDS), especially at the time of extracorporeal membrane oxygenation (ECMO) decision, is whether delivery might substantially improve the mother’s and child’s conditions. This multicenter, retrospective cohort aims to report maternal and fetal short- and long-term outcomes of pregnant women with ECMO-rescued severe ARDS according to the timing of the delivery decision taken before or after ECMO cannulation.

**Methods:**

We included critically ill women with ongoing pregnancy or within 15 days after a maternal/child-rescue-aimed delivery supported by ECMO for a severe ARDS between October 2009 and August 2021 in four ECMO centers. Clinical characteristics, critical care management, complications, and hospital discharge status for both mothers and children were collected. Long-term outcomes and premature birth complications were assessed.

**Results:**

Among 563 women on venovenous ECMO during the study period, 11 were cannulated during an ongoing pregnancy at a median (range) of 25 (21–29) gestational weeks, and 13 after an emergency delivery performed at 32 (17–39) weeks of gestation. Pre-ECMO PaO_2_/FiO_2_ ratio was 57 (26–98) and did not differ between the two groups. Patients on ECMO after delivery reported more major bleeding (46 vs. 18%, *p* = 0.05) than those with ongoing pregnancy. Overall, the maternal hospital survival was 88%, which was not different between the two groups. Four (36%) of pregnant women had a spontaneous expulsion on ECMO, and fetal survival was higher when ECMO was set after delivery (92% vs. 55%, *p* = 0.03). Among newborns alive, no severe preterm morbidity or long-term sequelae were reported.

**Conclusion:**

Continuation of the pregnancy on ECMO support carries a significant risk of fetal death while improving prematurity-related morbidity in alive newborns with no difference in maternal outcomes. Decisions regarding timing, place, and mode of delivery should be taken and regularly (re)assess by a multidisciplinary team in experienced ECMO centers.

**Supplementary Information:**

The online version contains supplementary material available at 10.1186/s13054-022-04189-5.

## Background

Acute respiratory distress syndrome (ARDS) is one of the main causes of non-obstetric mortality during pregnancy [1]. Physiological changes in maternal respiratory function and immune system during pregnancy make parturient women at higher risk of severe forms of respiratory failure, with ARDS occurring up to 70 in 100,000 pregnancies [2]. Various conditions can lead to ARDS during pregnancy, but most cases are associated with infectious pneumonia. Beyond the specific treatment of ARDS etiology, care of critically ill pregnant women with ARDS consists of protective mechanical ventilation, prone positioning when feasible, and conservative fluid administration strategies [2]. Venovenous extracorporeal membrane oxygenation (VV-ECMO) is currently used in the most severe patients with profound hypoxemia or high thoracic pressures refractory to lung-protective mechanical ventilation and prone position. In this severe population with ECMO, maternal mortality remains high (23–40%) with a significant incidence of fetal loss (23%) and perinatal complications [3]. Although rarely addressed in the literature, a key question in the care of critically pregnant women, especially at the time of the ECMO decision, is whether delivery might substantially improve the mother’s and child’s conditions. Few case series suggest that delivery of pregnant women suffering from respiratory failure could improve maternal conditions [4]. On the other hand, a delivery decision for maternal rescue often goes along with preterm birth which is the main cause of neonatal morbidity and mortality [5] and the second cause of death in children younger than 5 years of age [6]. Preterm birth ranges from extreme prematurity (< 28 weeks completed gestation) to late prematurity (34–37 weeks).

To date, few data are available to guide the management of critically ill pregnant women rescued by VV-ECMO. Besides, no literature was found on the influence of delivery decision timing, before or after ECMO cannulation, on both maternal and fetal outcomes.

The objectives of this multicenter, retrospective cohort were (a) to report maternal and fetal outcomes of pregnant women with ECMO-rescued severe ARDS, (b) to describe their critical care management and in-ICU complications, (c) to report long-term maternal health-related quality of life (HRQL) and long-term newborn outcomes and (d) to assess potential association between delivery-decision timing before or after ECMO cannulation and maternal and fetal outcomes.

## Methods

*Study design and patients* The study included critically ill pregnant women supported by ECMO for severe ARDS in four university tertiary intensive care units (ICU) between October 2009 and August 2021. Those four centers (Pitié-Salpétrière [Paris], Nancy hospital [Nancy], Pontchaillou hospital [Rennes], and North hospital [Marseille]) are the largest ECMO centers in France. All women with ARDS supported with ECMO during an ongoing pregnancy or in the early postpartum period were retrospectively included. The early postpartum period was defined as less than 15 days after a delivery decided for maternal and newborn rescue by a multidisciplinary expert team. Exclusion criteria were termination of pregnancy for maternal choice, first-trimester pregnant women or ECMO for isolated cardiogenic shock (i.e., cardiac arrest, pulmonary embolism, amniotic embolism, or anaphylactic shock). Included patients were then classified into two groups according to the time of ECMO, occurring either during an ongoing viable pregnancy or in the early post-partum period (< 15 days) following a maternal/child-rescue-aimed delivery.

According to the French research methodology MR004, this work was considered a non-interventional retrospective study and therefore only needed information from the survivors. The National Commission for Informatics and Liberties (CNIL) approved this study (no.1950673). Survivors gave oral consent to participate in the telephone interview conducted by the same investigator (S. AJ).

*Data collection* Clinical characteristics of the patients included Acute Physiology Score (SAPS) 2, Sepsis-Related Organ Failure Assessment (SOFA) scores, age, body mass index, comorbidities, and pregnancy-related complications. In addition, we collected pre-ECMO implantation information: previous rescue therapies, ventilator settings, arterial blood-gas parameters, Respiratory Extracorporeal Membrane Oxygenation Survival Prediction (RESP) score [7], and inotrope score. This latter was defined as the dose of dobutamine (μg/kg/ min) + (dose of epinephrine [μg/kg/min] + dose of norepinephrine [μg/kg/min]) × 100, and driving pressure was defined as plateau pressure minus positive end-expiratory pressure (PEEP). Gestational age at ECMO onset (i.e., in the ongoing pregnancy group) or at the time of emergency delivery (i.e., in the post-delivery ECMO group) was reported.

*ECMO management* An expanded data set including mechanical ventilation settings and adjuvant therapies on ECMO was noted. ECMO-related complications and organ dysfunction included clogged circuit/membrane, ECMO-circuit change, major bleeding, ischemic or hemorrhage stroke, renal replacement therapy, proven pulmonary embolism, pneumothorax, ventilator-associated pneumonia, and bacteremia. Major bleeding was defined as requiring ≥ 2 units of packed red blood cells for an obvious hemorrhagic event, necessitating a surgical or interventional procedure, or as an intracerebral hemorrhage or a fatal one. Patient management under venovenous (VV) ECMO support followed the Extracorporeal Life Support Organization guidelines [8]. Ultra-lung protective ventilation, following the settings of the ECMO arm of the EOLIA trial [9] was applied to all patients. Similarly, anticoagulation on VV-ECMO for non-COVID patients followed the EOLIA trial. In patients with COVID-19-related ARDS, the targeted activated partial thromboplastin time (aPTT) for anticoagulation of VV-ECMO with unfractionated heparin was increased to 60–75 s or anti-Xa activity 0.3–0.5 IU/mL (respective values were 40–55 s or 0.2–0.3 IU/mL in the EOLIA trial).

*Outcomes variables* Additional hospital outcomes aside from in-hospital maternal and newborn survival included the time spent on ECMO, mechanical ventilation, in ICU, and in the hospital, respectively. Obstetrical and fetal outcomes included birth terms, type of delivery, birth weight, newborn survival at day 1, and need for neonatal ICU.

*Long-term outcome variable* Patients alive in March 2022 were evaluated for their HRQL psychological and physical status during a phone call interview explaining the purpose and objectives of the study. They completed the French version of the validated Short-Form 36 (SF-36; based on the Medical Outcome Study Survey) questionnaire. Its standardized combination of 36 items evaluates eight domains (physical functioning, role-physical, bodily pain, general health, vitality, social functioning, role-emotional, and mental health) and results were computed as recommended by developers [10, 11]. Our patients’ mean SF-36 levels were compared with those obtained for French age- and sex-matched controls with no adverse health conditions. We also screened for post-traumatic stress disorder (PTSD)-related symptoms, completing the Impact of Event Scale-Revised (IES-R) questionnaire, with a score ≥ of 33 indicating a likely diagnosis of PTSD [12, 13]. Other long-term maternal outcomes included work resumption and new pregnancy occurrence. Newborn long-term outcomes were also retrospectively assessed during that interview and combined occurrence of any severe complications of premature birth (i.e., intraventricular hemorrhage, bronchopulmonary dysplasia, necrotizing enterocolitis, retinopathy, or cerebral palsy), child health status and neurological development at follow-up time.

*Statistical analyses* This study followed CONSORT recommendations for reporting cohort studies (STROBE statement) [14]. Data are reported as percentages for categorical variables and medians (range) for continuous variables. Continuous data were assessed for normal distribution by the Shapiro–Wilk test. Group comparison was performed using Student’s t or Mann–Whitney tests, as appropriate. Statistical analyses were computed with StatView and Prism software. All tests were two-tailed with *p* < 0.05 being considered statistically significant.

## Results

*Study population* During that 12-year study period, 1529 patients with refractory ARDS eligible for ECMO were screened to identify 478 women of whom 83 were pregnant or early postpartum patients. Among eligible patients, ten were excluded because of post-abortion context, five because of ectopic pregnancy, and forty-four because ARDS complicated the evolution of an initial cardiogenic shock or cardiac arrest. Finally, twenty-four patients were included, with, respectively, 11 patients receiving ECMO during ongoing pregnancy and 13 early after delivery (Additional file 1).

Patients’ pre-ECMO characteristics according to their pregnancy status at the time of ECMO onset are reported in Table 1. Briefly, their median (range) age, BMI, and SAPS II were 33 (19–42) years, 33 (21–47) kg/m^2^, and 54 (17–78), respectively. Pre-ECMO comorbidities were not different between patients on ECMO during ongoing pregnancy and those cannulated after an emergency delivery. The main cause of ARDS was viral pneumonia (Influenza H_1_N_1_ or COVID-19) in both groups. Similarly, pre-ECMO ventilation parameters, blood gas, and respiratory mechanics did not differ between the two groups, except for higher pre-ECMO respiratory rates in patients on ECMO after delivery. Noticeably, pre-ECMO PaO_2_/FiO_2_ was 57 (26–98) and only 5 (45%) and 8 (62%) of patients in the two groups received prone positioning before ECMO onset. Lastly, the median (range) gestational age at ECMO cannulation was 25 (21–29) weeks in patients cannulated during ongoing pregnancy with a fetus weight estimated at 828 (250–1390) g. In the other group, delivery was performed after 32 (17–39) weeks of gestation and ECMO started 6 (0–14) days after delivery. The primary setting was venovenous (VV) ECMO for all patients except one patient in each group who required veno-arterial (VA) ECMO because of influenza-associated cardiomyopathy.

**Table 1:** Patients’ pre-ECMO characteristics according to their pregnancy status on ECMO

	All (*N* = 24)	Ongoing pregnancy on ECMO (*N* = 11)	Delivery before ECMO (*N* = 13)	*p*
Age, years	33 (19–42)	33 (19–42)	32 (25–39)	0.87
Body mass index, kg/m^2^	33 (21–47)	31 (21–43)	35 (23–47)	0.25
SAPS II score	54 (17–78)	51 (17–75)	57 (45–78)	0.40
SOFA score	11 (4–16)	11 (4–16))	11 (8–16))	0.88
RESP score	6 (3–7)	6 (3–7)	6 (4–7)	0.43
Hypertension	2 (8)	1 (9)	1 (8)	0.90
Diabetes mellitus	2 (8)	1 (9)	1 (8)	0.90
Asthma	3 (13)	2 (18)	1 (8)	0.44
*ARDS etiology*				
Viral pneumonia	22 (92)	9 (82)	13 (100)	0.10
Influenza H_1_N_1_	9 (38)	3 (18)	6 (46)	0.34
Sars-Cov-2	13 (54)	6 (55)	7 (54)	0.97
Bacterial pneumonia	1 (4)	1 (9)	0 (0)	0.27
Other	1 (4)	1 (9)	0 (0)	0.27
*Pregnancy details*				
Gestational diabetes	7 (29)	3 (27)	4 (31)	0.20
Pre-eclampsia	1 (4)	1 (9)	0 (0)	0.27
Gestational age at ECMO onset, weeks	–	25 (21–29)	–	–
Child term at the delivery decision, weeks	–	–	32 (17–39)	–
*Time from*				
Intubation to ECMO, days	3 (0–15)	3 (0–13)	4 (0–15)	0.41
Delivery to ECMO, days	–	–	6 (0–14)	–
*Ventilation parameters*				
FiO_2_, %	100 (100–100)	100 (100–100)	100 (100–100)	1
PEEP, cm H_2_O	12 (6–20)	13 (7–18)	11 (6–20)	0.45
Tidal volume, mL/kg PBW	6.5 (5–10)	6.7 (5.0–10)	6.3 (5.8–7.3)	0.26
Respiratory rate, no./min	31 (16–40)	27 (16–32)	34 (28–40)	< 0.01
Driving pressure, cm H_2_O	18 (8–25)	17 (14–21)	19 (8–25)	0.24
*Last blood-gas values pre-ECMO*				
pH	7.24 (6.91–7.37)	7.25 (6.99–7.37)	7.22 (6.91–7.35)	0.73
PaO_2_/FiO_2_	57 (26–98)	58 (38–70)	56 (26–98)	0.57
PaCO_2_, mm Hg	59 (34–100)	54 (34–100)	64 (46–97)	0.18
Arterial lactate, mmol/L	3.5 (0.8–17)	3.5 (1–16)	3.5 (0.8–17)	0.97
*Rescue therapy pre-ECMO*				
Neuromuscular blockade	22 (92)	9 (91)	13 (100)	0.26
Prone positioning	13 (54)	5 (45)	8 (62)	0.56
Inhaled nitric oxide	6 (25)	2 (18)	4 (31)	0.48
Recruitment maneuvers	4 (17)	3 (27)	1 (8)	0.20
*Pre-ECMO complications*				
Pneumothorax	3 (13)	1 (9)	2 (15)	0.64
Bacterial co-infection	4 (17)	1 (9)	3 (23)	0.77
Pulmonary embolism	1 (4)	0 (0)	1 (8)	0.35
Inotrope score, μg/kg/min	36 (0–235)	42 (0–235)	30 (0–77)	0.68

Data are expressed as *n* (%) or median (range)

*ECMO* extracorporeal membrane oxygenation; *SAPS II* Simplified Acute Physiology Score; *SOFA* Sequential Organ Failure Assessment; *RESP* Respiratory Extracorporeal Membrane Oxygenation Survival Prediction; *ARDS* Acute respiratory distress syndrome; *PEEP* Positive End Expiratory Pressure; *PBW* Predicted Body Weight

**Fig. 1:**
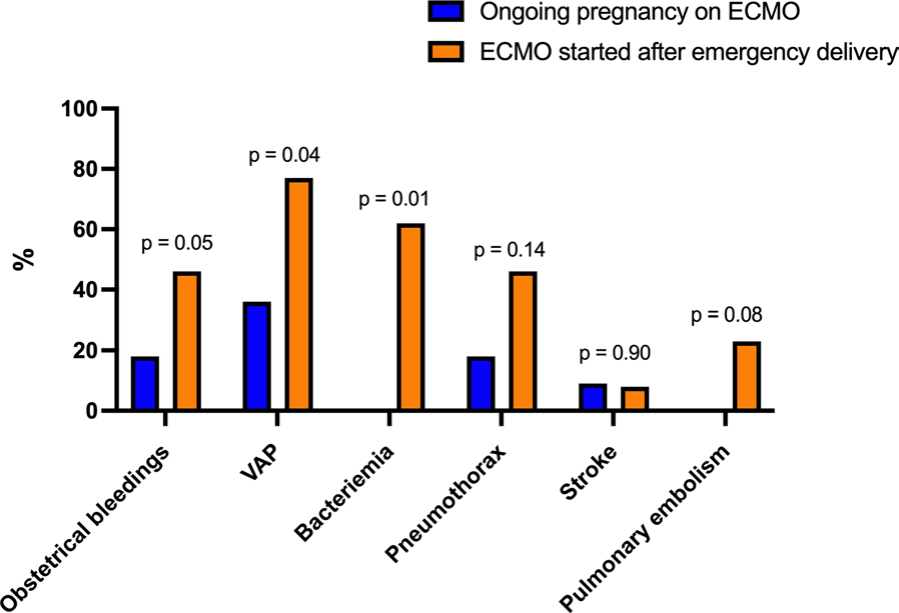
Main in-ICU complications in critically ill pregnant or peripartum women with severe ARDS supported by ECMO according to the timing of delivery. ARDS, Acute respiratory distress syndrome; ECMO, extracorporeal membrane oxygenation; ICU, intensive care unit; VAP, ventilator-associated pneumonia

*ECMO and in-ICU complications* Among in-ICU complications reported in Table 2 and Fig. 1, bleedings occurred more frequently in the patients on ECMO after delivery, with a higher rate of obstetrical major bleeding (i.e., uterine bleedings, abdominal parietal hematoma, or hemoperitoneum) (46 vs. 18%, *p* = 0.05). A surgical revision was needed in one-third of those patients. Similarly, we reported a significantly higher rate of ventilator-associated pneumonia and bacteremia, and a more frequent change of ECMO circuit in this latter group due to membrane dysfunction, severe hemolysis, or high fibrinogen consumption. Other complications did not significantly differ between the two groups. In the group on ECMO after delivery, three patients were switched from VV-ECMO to veno-arteriovenous ECMO due to pulmonary embolism or viral myocarditis. The single patient with VA-ECMO was turned into VV-ECMO after 6 days of cardiac support. Neuromuscular blockades were administered in 10 (91%) pregnant patients and 11 (85%) post-partum patients. Prone positioning was performed in 3 (27%) pregnant women on ECMO, respectively, at 22, 24 and 26 weeks of gestation, and 5 (38%) ECMO-rescued post-partum women. Due to refractory hypoxemia on ECMO, one critically ill patient had hypothermia, and 2 (8%) received beta-blockers, with no difference according to the pregnancy status.

**Table 2:** Patients’ ECMO management and in-ICU complications according to their pregnancy status on ECMO

	All (*N* = 24)	Ongoing pregnancy on ECMO (*N* = 11)	Delivery before ECMO (*N* = 13)	*p*
*Type of ECMO support*				
Venovenous ECMO	22 (92)	10 (91)	12 (92)	0.90
Venoarterial ECMO	2 (8)	1 (9)	1 (8)	0.90
Switch from VV to VA	1 (4)	1 (9)	0 (0)	0.27
Switch from VV to V-AV	3 (13)	0 (0)	3 (23)	0.09
Switch from VA to VV	1 (4)	0 (0)	1 (8)	0.35
*ECMO-related complications*				
Clogged circuit/membrane requiring a change	1 (4)	1 (9)	0 (0)	0.26
At least one circuit change	10 (42)	2 (18)	8 (62)	0.03
*Major bleeding*				
Cannula bleeding	12 (50)	5 (45)	7 (54)	0.66
Obstetrical bleeding*	8 (33)	2 (18)	6 (46)	0.05
Number of RBC transfusions	7 (0–50)	7 (0–50)	7 (2–23)	0.11
Stroke	2 (8)	1 (9)	1 (8)	0.90
Pneumothorax on ECMO	8 (33)	2 (18)	6 (46)	0.14
*Acute kidney injury*				
KDIGO ≥ 2	9 (38)	3 (27)	6 (46)	0.34
Renal replacement therapy	9 (38)	2 (18)	6 (46)	0.14
≥ 1 antibiotic-treated VAP	14 (58)	4 (36)	10 (77)	0.04
≥ 1 antibiotic-treated bacteremia episode(s)	8 (33)	0 (0)	8 (62)	< 0.01
Tracheostomy	10 (42)	3 (27)	7 (54)	0.19
Tidal volume on ECMO Day 1, ml/kg PBW	3.2 (1.0–7.4)	3.1 (1.0–6.3)	3.2 (1.7–4.0)	0.93
*On ECMO*				
Neuromuscular blockade	21 (88)	10 (91)	11 (85)	0.64
Prone positioning	8 (33)	3 (27)	5 (38)	0.56
Inhaled nitric oxide	3 (13)	1 (9)	2 (15)	0.64
Hypothermia	1 (4)	1 (9)	0 (0)	0.26
Beta-blockers	2 (8)	1 (9)	1 (8)	0.90
Antenatal corticosteroid therapy	14 (58)	7 (63)	7 (54)	0.68

Data are expressed as *n* (%) or median (range)

*ECMO* extracorporeal membrane oxygenation; *ICU* intensive care unit; *VV* venovenous; *VA* venoarterial; *V-AV* veno-arteriovenous; *RBC* red blood cells; *KDIGO* Kidney Disease Improving Global Outcomes; *PBW* Predicted Body Weight

*Uterine bleedings or abdominal parietal hematoma or hemoperitoneum

*Maternal outcomes* Maternal survival rates at ICU discharge were similar in the 2 groups, with, respectively, 9 (82%) survivors in the group with ECMO during pregnancy and 12 (92%) among those cannulated after delivery (*p* = 0.44) (Table 3). These survival rates slightly decreased to 73 and 85% in the 2 groups at hospital discharge (*p* = 0.47). Maternal deaths were attributed to multiple organ failure on ECMO (*n* = 2), brain death (*n* = 1), post-lung transplant after 65 days on ECMO (*n* = 1), and septic shock one month after ICU discharge (*n* = 1). ECMO duration was significantly lower when ECMO started during ongoing pregnancy (20 [3–95] vs. 33 [5–71] days; *p* = 0.04), whereas mechanical ventilation duration and ICU length of stay were not statistically different between the two groups. Except for one fetal loss due to medical termination of pregnancy for maternal rescue purposes, all patients who delivered before ECMO had a c-section, whereas this procedure was performed in only 3 (27%) patients when ECMO was set during pregnancy. In this group, vaginal delivery occurred in 4 (36%) patients and 4 (36%) others had a spontaneous in-ICU expulsion during their ECMO run. The long-term evaluation was conducted after 61 (4–172) months. A low rate of patients returned to work in both groups (29% vs. 22%, *p* = 1). More than half of the patients cannulated during pregnancy, and one-third of those who received ECMO after delivery had a new pregnancy during the follow-up period. Long-term HRQL was still profoundly impaired with regular psychological counselling ongoing for five of them, whereas chronic fatigue and respiratory lasting sequelae still significantly affected work or daily activities. However, symptoms compatible with PTSD and HRQL did not differ according to the timing of ECMO onset during the pregnancy (Additional file 2).

**Table 3:** Maternal and newborn outcomes of critically ill women with refractory ARDS rescued by ECMO according to their pregnancy status on ECMO

	All (*N* = 24)	Ongoing pregnancy on ECMO (*N* = 11)	Delivery before ECMO (*N* = 13)	*p*
*Maternal outcomes*				
Delivery procedures				
C-section	15 (63)	3 (27)	12 (92)	< 0.01
Vaginal delivery	4 (17)	4 (36)	0 (0)	0.02
In-ICU expulsion*	5 (21)	4 (36)	1 (8)	0.13
General anesthesia or ICU sedation	18 (75)	8 (73)	10 (77)	0.81
Epidural anesthesia	6 (25)	3 (27)	3 (23)	0.81
ECMO duration, days	20 (3–95)	20 (3–95)	33 (5–71)	0.04
In survivors	26 (5–95)	25 (5–95)	27 (5–52)	0.24
Mechanical ventilation duration, days	42 (3–116)	37 (3–116)	47 (8–89)	0.47
In survivors	41 (8–116)	41 (9–116)	40 (8–71)	0.96
ICU length of stay, days	48 (7–120)	42 (7–120)	53 (16–115)	0.25
In survivors	46 (14–120)	46 (14–120)	45 (14–74)	0.92
ICU discharge survival	21 (88)	9 (82)	12 (92)	0.44
Hospital discharge survival	19 (79)	8 (73)	11 (85)	0.47
Long-term maternal outcomes^£^				
Time from ICU discharge to evaluation, months	61 (4–172)	56 (4–145)	65 (9–192)	0.76
Returned to work^££^	4 (25)	2 (29)	2 (22)	1
IES revised score	30 (12–51)	29 (12–51)	31 (12–49)	0.91
PTSD**	6 (38)	3 (43)	3 (33)	0.70
Other pregnancies	7 (44)	4 (57)	3 (33)	0.34
*Newborn outcomes*				
Estimated fetus weight at ECMO onset, g	–	828 (250–1390)	–	–
Birth term, weeks	33 (23–39)	31 (23–38)	34 (25–39)	0.47
Alive at birth	18 (75)	6 (55)	12 (92)	0.03
Alive on Day 1 post-delivery	16 (67)	5 (45)	11 (85)	0.04
Birth weight, g	2157 (500–4000)	1733 (500–2750)	2580 (600–4000)	0.14
Stay in neonatal ICU	7 (39)	2 (33)	5 (42)	0.35
Severe preterm morbidity in survivors***	0 (0)	0 (0)	0 (0)	1
Healthy child at the time of follow-up	16 (67)	5 (45)	11 (85)	0.04

Data are expressed as *n* (%) or median (range)

*ECMO* extracorporeal membrane oxygenation; *ARDS* acute respiratory distress syndrome; *ICU* intensive care unit; *IES* Impact of Event Scales; *PTSD* post-traumatic stress disorder

*Spontaneous in-ICU expulsion on ECMO in the ongoing pregnancy group or expulsion before ECMO onset in the “delivery before ECMO” group with 1 fetal loss due to medical termination of the pregnancy for maternal rescue purposes at 17 weeks of gestation

**Score ≥ 33 at the revised Impact of Event Scale (IES-r) being compatible with a probable diagnosis of PTSD

***Intraventricular hemorrhage, bronchopulmonary dysplasia, necrotizing enterocolitis, retinopathy, cerebral palsy

£Phone call interview was performed on 7 survivors from the “ongoing pregnancy on ECMO” group and 9 survivors from the “delivery before ECMO” group

££Respectively, 1 in 7 and 2 in 9 survival women in the “ongoing pregnancy on ECMO” and “delivery before ECMO” groups did not work before hospitalization

**Fig. 2:**
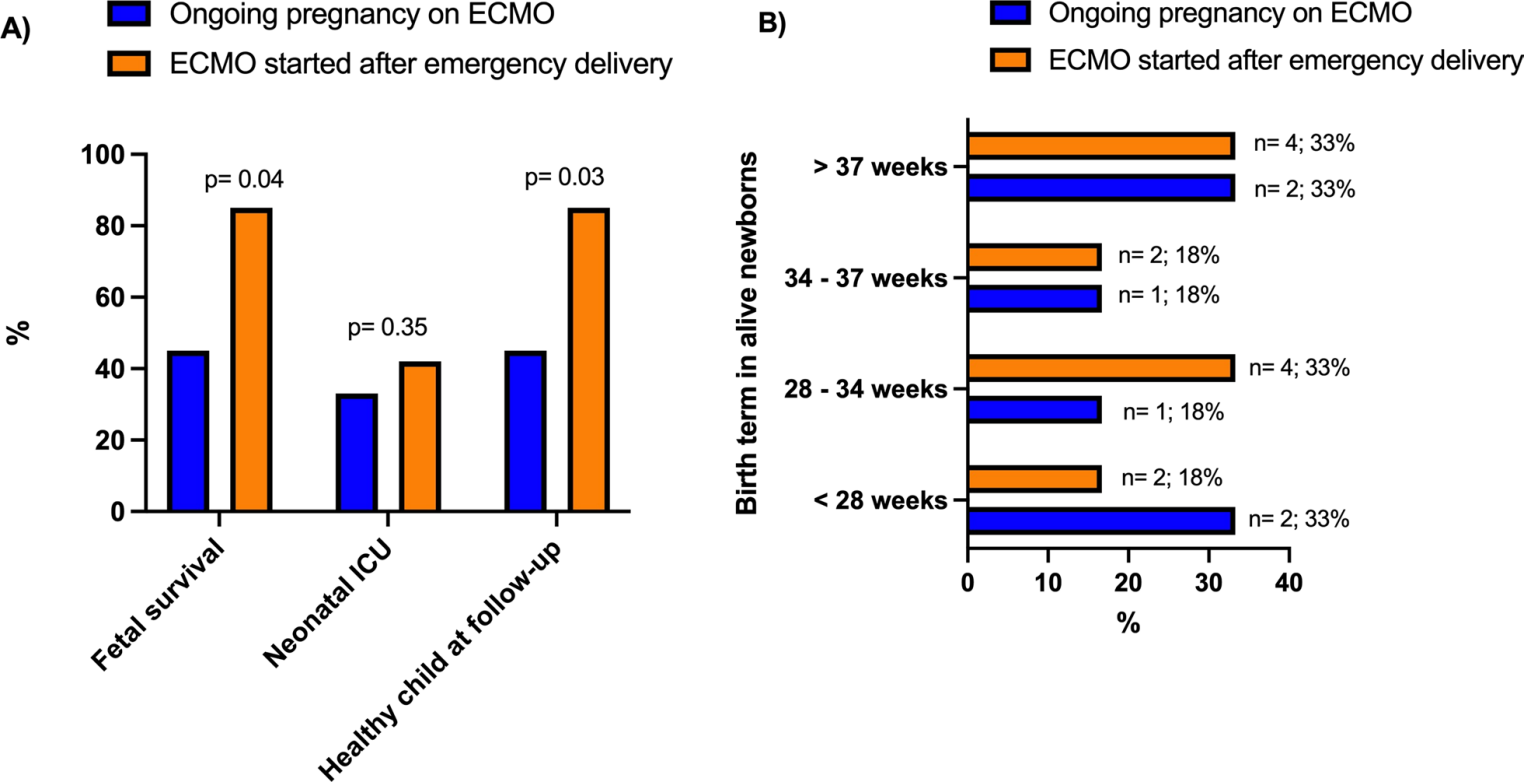
Childbirth survival **A** and birth terms **B** in critically ill pregnant or peripartum women with severe ARDS supported by ECMO according to the timing of delivery. ARDS, Acute respiratory distress syndrome; ECMO, extracorporeal membrane oxygenation; ICU, intensive care unit

*Newborn outcomes* Six (55%) newborns were alive at birth after 31 (23–38) weeks of gestation when ECMO was set during pregnancy. Survival was significantly higher when ECMO was set after delivery (92%, *p* = 0.03). One newborn in each group died within 24 h, and the need for neonatal intensive care was similar between the two groups. Two (33%) newborns had extreme prematurity (< 28 weeks) when pregnancy was pursued on ECMO, whereas they were 2 (18%) when ECMO was started after delivery (Fig. 2). Among survivors, no severe preterm morbidity was reported in both group and children’s health and neurological development abilities were considered normal after a median follow-up of 61 (9–145) months.

## Discussion

Herein, we described a case series of critically ill pregnant or peripartum women who received ECMO support for severe ARDS with an original focus on delivery-decision timing and maternal and fetal outcomes. As the decision of ending a preterm viable pregnancy for maternal rescue purposes often goes along with high morbidity and mortality risks in fetuses and newborns, one could legitimately argue that carried-on pregnancies for additional weeks on ECMO support might lead to better outcomes. Despite an extreme initial severity, we report a high maternal survival rate (> 70%) with no difference according to the decision of delivery before ECMO onset and a lower long-term newborn survival rate when pregnancy was pursued on ECMO compared to those urgently delivered before ECMO (45 vs. 85%, *p* = 0.04). However, different gestation terms at the time of the ECMO decision could explain this latter finding.

The risk of developing severe viral illnesses is increased in pregnant patients compared with their no pregnant counterparts, particularly if they contract the infection in the third trimester of pregnancy [15]. These increased risks in pregnancy have become greater with successive variants of SARS-CoV-2 and were the highest for the delta variant [16]. In our cohort, nearly all patients had viral pneumonia (influenza or COVID-19). Noticeably, only 13 (54%) of our patients received prone positioning before ECMO which contrasts with the high rates of prone positioning usually reported in French ICUs, especially during the COVID-19 pandemic [17, 18]. Data about the prone position in pregnant women are limited to case reports but are reassuring. Routine indications and contraindications should apply, although it may be appropriate to avoid the position in the immediate postoperative period after cesarean birth. In a case series of obstetric patients with severe COVID-19-related ARDS, 13 patients had prone positioning. None proning sessions were terminated owing to maternal hemodynamic or respiratory instability, or fetal intolerance [19].

High survival with ECMO has been reported in pregnant and peripartum women with COVID-19 in the Extracorporeal Life Support Organization Registry [20]. Among 1180 adult female patients supported with VV-ECMO for COVID-19, the 100 pregnant or peripartum women were more likely to survive hospital discharge (84% vs. 51.5%; overlap propensity score weighted OR 1.18; 95% CI 1.10–1.27) and had fewer renal complications related to ECMO. However, the proportion of active pregnancies or postpartum patients, obstetrical terms, the fetal loss rate on ECMO, birth terms and neonatal outcomes according to gestational age or preterm delivery decision at cannulation were not reported in this large cohort [20]. Current consensus recommendations from the UK suggest that ECMO indication should be undertaken with the same criteria as for non-pregnant patients [21]. However, anticoagulation management in this specific population could be challenging during ECMO. High inflammatory syndrome, pregnancy, and COVID-19 are all known over-risks of venous thrombosis [22, 23] and may suggest targeting a higher anticoagulation regimen on ECMO compared to non-pregnant women. On the other hand, we observed a higher rate of obstetrical bleeding in patients cannulated after delivery which reinforces the need to get a complete assessment of hemostasis in these patients with a high risk of bleeding. As the normal serum level of fibrinogen in third-trimester pregnant women rises to 5 g/L [24], a more liberal target fibrinogen level of at least 2–2,5 g/dL could be applied in parturient on ECMO. Because ECMO-induced hypothermia results in altered primary hemostasis, normothermia must be obtained. Stillbirth can be associated with disseminated induced coagulopathy and prompt delivery is recommended in case of late intrauterine fetal death on ECMO. Obstetricians must also ensure complete placenta delivery in case of spontaneous expulsion on ECMO. Although the impact of the cannulation site on obstetrical bleeding has not been thoroughly studied yet, one may argue that a single-site dual lumen right internal jugular cannulation might be considered in case of uncontrolled obstetrical bleeding exacerbated by increased abdominal venous pressure.

Improvement of maternal respiratory function following delivery remains unclear in the literature [4]. Pregnancy can increase oxygen consumption by up to one-third, reduce respiratory system compliance and functional residual capacity, impair gas exchanges, and increase the respiratory drive with progesterone rise [25]. Besides, maternal arterial oxygen saturation, PaO_2_, PaCO_2_, hemoglobin level, and cardiac output need to be optimized and closely monitored to ensure adequate fetal oxygenation [26]. Tomlinson et al. [27] reported that emergent delivery in the context of acute respiratory failure could lower by 28% FiO_2_ needs. Other observational studies emphasized that ARDS mortality during pregnancy did not significantly differ from that in non-pregnant patients [3]. Our results concur with this latter point, with no shorter time on ECMO nor on mechanical ventilation in patients for whom ECMO was started after delivery. These promising maternal outcomes should not overshadow that their long-term HRQL was still seriously altered compared with the general French population and ECMO-rescued ARDS survivors, especially in the physical, general health, role emotional and mental health domains.

However, fetal outcomes significantly differed according to the timing of ECMO onset before or after delivery with 45% of stillbirths and 36% of in-ICU spontaneous expulsions at viable terms in our ECMO-rescued pregnant patients. It contrasts with 8% newborn mortality rates when the delivery decision was taken before ECMO started. These results concur with a 35% fetal mortality rate reported in a review of 26 studies including 41 pregnant women (mean gestational age of 26 weeks) with ECMO-supported ARDS [3]. Among our 11 patients with ongoing pregnancy on ECMO, 10 fetuses already reached the “theoretical” viability criteria at the time of ECMO onset (≥ 23 weeks and ≥ 500 g). In the French EPIPAGE-2 prospective cohort study, the fetal disability-free survival rate at 25 weeks of gestation (median term when the decision was taken to pursue the pregnancy on ECMO and to postpone delivery in our cohort) is estimated at 30% [28]. Prolonging intra-uterine time with ECMO support allowed to double fetus weights and might have reduced neonatal severe complications and long-term handicaps in child survivors. Compared with children born before ECMO at a median term of 34 weeks, we did not report a higher rate of neonatal ICU stay nor long-term disabilities among alive newborns after prolonged weeks of gestation on ECMO. As prognosis factors in premature births are gestational age, birth weight, prenatal administration of corticoids, maternal infection context, and neonatal intensive care resources [29], continuing the pregnancy on ECMO support should be balanced with the high morbidity and mortality rates linked to extreme premature birth [29–33]. Since critically ill mothers on ECMO are at high risk of preterm delivery in case of fetal distress, antenatal corticoid therapy for fetal lung maturation should be widely used in women from 24 to 34 weeks of gestation with no current infection. Fetal neuroprotection before 32 weeks with MgSO_4_ should be individualized considering potential side effects including pulmonary edema and cardiovascular toxicity. In both cases, antenatal prophylaxis should not delay urgent delivery if required. It should also involve daily fetal monitoring and frequent reevaluation of the delivery timing with a multidisciplinary team including obstetricians, neonatologists, and intensivists.

We are aware that our study has limitations. First, because of its retrospective design, our study cannot raise a robust association between maternal/fetal outcomes and the timing of ECMO and delivery. Besides, baseline gestational age differences at the time of the ECMO start may likely explain unadjusted fetal outcomes. Second, pregnant women or post-partum women represent less than 5% of the women on ECMO for severe ARDS. Therefore, our sample size was limited. Third, we might have missed some long-term cognitive sequelae, as we did not thoroughly evaluate the cognitive functions of the children with validated tests. Fourth, patients were included for 12 years. Thus, we cannot rule out that ECMO management has not changed during the period of the study. Fifth, our population was not compared with pregnant women with severe ARDS who did not receive ECMO. Lastly, 22 out of 24 cases were secondary to influenza or COVID-19 pneumonia, with potential direct fetal effects of the viruses [34, 35].

## Conclusion

Management of critically ill pregnant women with refractory ARDS supported by ECMO raises the challenging question of optimal delivery timing. Continuation of the pregnancy on ECMO support carries a significant risk of fetal death while being associated with fewer prematurity-related morbidity in alive newborns and no difference in maternal outcomes. Decisions regarding timing, place, and mode of delivery should be taken and regularly (re)assess by a multidisciplinary team including obstetricians, neonatologists, and intensivists experienced in the care of ECMO in pregnancy. Ideally, this should take place in ECMO centers with experience and expertise in all these specialities.

## Supplementary Information

**Additional file 1** Study flowchart. ECMO, extracorporeal membrane oxygenation; VV, veno venous; ARDS, acute respiratory distress syndrome.

**Additional file 2** Comparison of health-related quality of life assessed by mean SF-36 scores in ECMO-rescued ARDS survivors according to the timing of delivery. ECMO, extracorporeal membrane oxygenation; ARDS, acute respiratory distress syndrome; ICU, intensive care unit; IES, impact of event scales; PTSD, post-traumatic stress disorder.

## Data Availability

The datasets used during the current study are available from the corresponding author on reasonable request.

## Abbreviations

ARDS: Acute respiratory distress syndrome
ICU: Intensive care unit
HRQOL: Health-related quality of life
PTSD: Post-traumatic stress disorder
SAPS II: Simplified Acute Physiology Score II
SOFA: Sequential Organ-Failure Assessment
BMI: Body mass index
VV-ECMO: Venovenous extracorporeal membrane oxygenation
VA-ECMO: Venoarterial extracorporeal membrane oxygenation
PEEP: Positive End Expiratory Pressure
PBW: Predicted Body Weight
IES-R: Impact of Event Scale-Revised
RESP: Respiratory Extracorporeal Membrane Oxygenation Survival Prediction
aPTT: Activated partial thromboplastin time
KDIGO: Kidney Disease Improving Global Outcomes
RBC: Red blood cells
VAP: Ventilator-associated pneumonia
