# Supplementary material for: Delivery decision in pregnant women rescued by ECMO for severe ARDS: a retrospective multicenter cohort study
Source: Crit Care. 2022 Oct 17;26:312. doi: 10.1186/s13054-022-04189-5 (PMC9574812; doi:10.1186/s13054-022-04189-5)
Supplement: Supplementary file 1 — Additional file 1 Study flowchart. ECMO, extracorporeal membrane oxygenation; VV, veno venous; ARDS, acute respiratory distress syndrome. [file 13054_2022_4189_MOESM1_ESM.docx]

**Supplementary File 1. Study Flowchart.**

*ECMO, extracorporeal membrane oxygenation; VV, veno venous; ARDS, acute respiratory distress syndrome.*
