# Supplementary material for: Delivery decision in pregnant women rescued by ECMO for severe ARDS: a retrospective multicenter cohort study
Source: Crit Care. 2022 Oct 17;26:312. doi: 10.1186/s13054-022-04189-5 (PMC9574812; doi:10.1186/s13054-022-04189-5)

**Supplementary File 2. Comparison of health-related quality of life assessed by mean SF-36 scores in ECMO-rescued ARDS survivors according to the timing of delivery.**


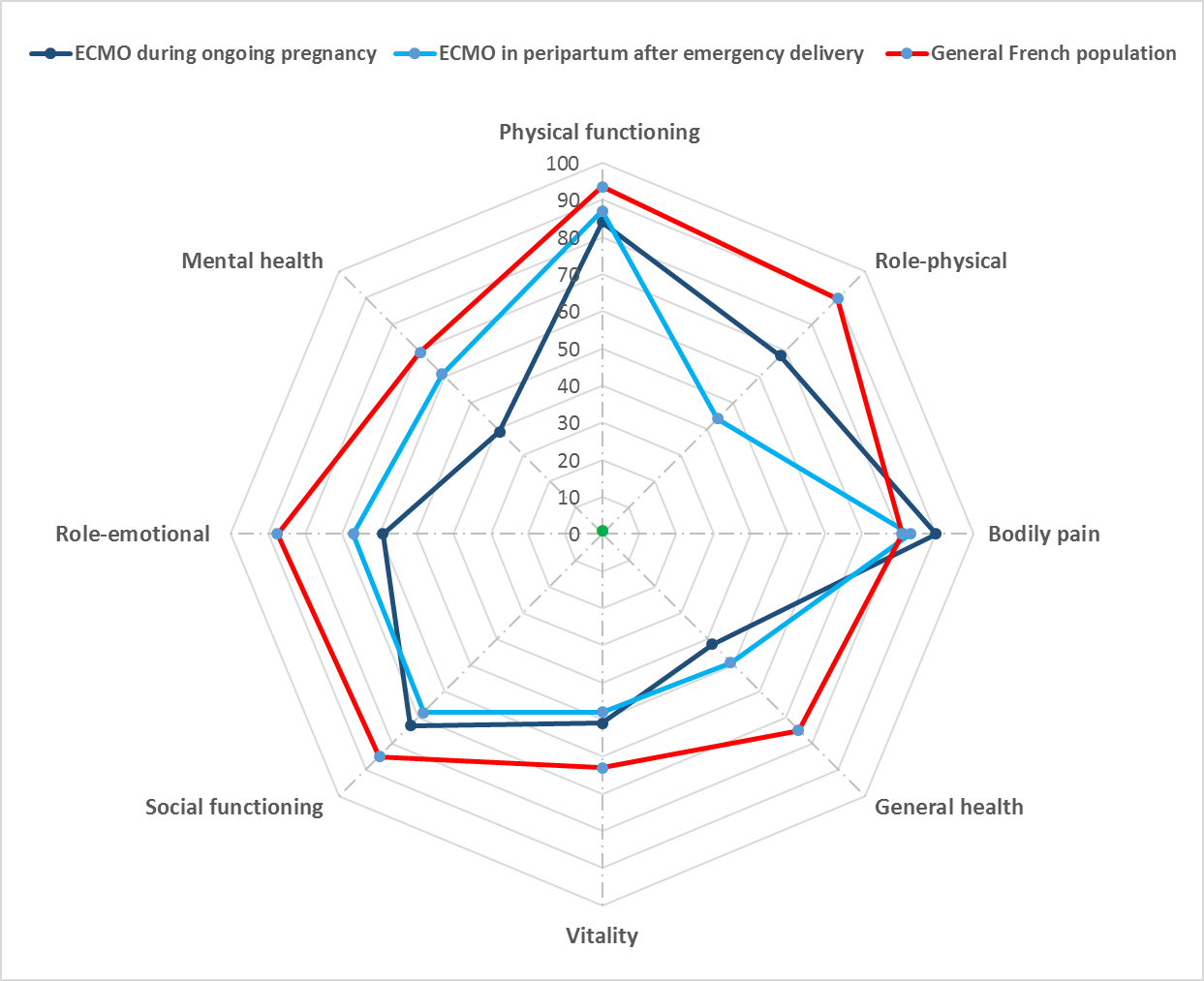

Supplement: Supplementary file 2 — Additional file 2 Comparison of health-related quality of life assessed by mean SF-36 scores in ECMO-rescued ARDS survivors according to the timing of delivery. ECMO, extracorporeal membrane oxygenation; ARDS, acute respiratory distress syndrome; ICU, intensive care unit; IES, impact of event scales; PTSD, post-traumatic stress disorder. [file 13054_2022_4189_MOESM2_ESM.docx]
